# Case Report: Adult EBV-associated hemophagocytic lymphohistiocytosis with presumed primary EBV infection: a report of three cases and diagnostic considerations

**DOI:** 10.3389/fmed.2026.1950374

**Published:** 2026-10-07

**Authors:** Ayuri Ishikawa, Hiroshi Yasui, Sanshiro Uchida, Kumi Oshima, Fumiaki Sano, Ayako Arai

**Affiliations:** 1 Department of Hematology and Oncology, St. Marianna University School of Medicine, Kawasaki, Japan; 2 The Institute of Medical Science, The University of Tokyo, Tokyo, Japan; 3 Department of Hematology, Graduate School of Medical and Dental Sciences, Institute of Science Tokyo, Tokyo, Japan; 4 Department of Hematology and Biophysical Systems Analysis, Graduate School of Medical and Dental Sciences, Institute of Science Tokyo, Tokyo, Japan

**Keywords:** disease stratification, EBV-associated HLH, epstein–barr virus, hemophagocytic lymphohistiocytosis, primary EBV infection, stem cell transplantation

## Abstract

Adult Epstein–Barr virus-associated hemophagocytic lymphohistiocytosis (EBV-HLH) is a clinically heterogeneous syndrome comprising biologically distinct disease entities. We report three adults with EBV-HLH and describe their diagnostic evaluation and clinical course. We presumed primary EBV infection from anti-VCA IgM positivity, anti-EBNA negativity, and a history of possible saliva-mediated exposure in all three patients. Personal and family histories did not suggest familial HLH (fHLH), and genetic analysis performed in one patient did not identify pathogenic variants of fHLH. No history nor clinical findings suggested an inborn error of immunity. Whole-body computed tomography did not show any mass lesions suggestive of malignancy, and bone marrow examination showed hemophagocytosis without abnormal lymphoid proliferation or other findings suggestive of lymphoma. No clonal immunoglobulin heavy-chain or T-cell receptor gene rearrangements were detected in any patient. EBV-infected cell phenotype was assessed by bone marrow pathology in two patients; EBER-positive CD3-positive T cells were identified in one, whereas the infected cell phenotype could not be determined in the other because of the limited number of cells. In the remaining patient, EBV-DNA quantification in sorted peripheral blood mononuclear cell fractions showed EBV infection of CD8-positive T cells. All patients responded to HLH-94/HLH-2004-based therapy including etoposide and were managed without hematopoietic stem cell transplantation (HSCT). During follow-up at our department for 345–1,067 days, no confirmed HLH recurrence was observed, although EBV-DNA remained detectable for more than one year in two patients. These cases suggest that EBV-DNA may remain detectable after clinical remission in adult HLH associated with presumed primary EBV infection. The indication for HSCT should be considered carefully, not on the basis of EBV-DNA positivity alone, but through longitudinal clinical assessment and reassessment for CAEBV, EBV-positive lymphoma, fHLH, and underlying immunodeficiency.

## 1 Introduction

Hemophagocytic lymphohistiocytosis (HLH) is a life-threatening hyperinflammatory syndrome characterized by excessive activation of cytotoxic lymphocytes, natural killer cells, and macrophages (1). Epstein–Barr virus-associated HLH (EBV-HLH) is clinically heterogeneous and may arise in the setting of presumed primary EBV infection, occult or overt immune disorders, chronic active EBV disease (CAEBV), or EBV-positive lymphomas (2–5). These underlying conditions are considered to differ in prognosis and treatment requirements. However, studies systematically comparing the clinical features and the outcomes of EBV-HLH according to underlying disease category remain limited. Stratification according to the underlying disease context is essential to determine the appropriate treatment strategies and to avoid unnecessary intensive treatment, including hematopoietic stem cell transplantation (HSCT), particularly in younger patients. Although HSCT may be effective for familial, refractory, recurrent, or lymphoma-associated HLH, some cases related to primary EBV infection may not require HSCT and may follow a more favorable course (6, 7). Here, we describe three adults with EBV-HLH in the setting of presumed primary EBV infection who were successfully managed without HSCT.

## 2 Case presentation

We retrospectively reviewed adult patients diagnosed with EBV-HLH at St. Marianna University Hospital between 2018 and 2025. HLH was diagnosed according to the HLH-2004 criteria (8). Presumed primary EBV infection was determined by EBV serology, including anti-VCA IgM positivity and anti-EBNA negativity, with acute clinical presentation, disease course, and recent history compatible with possible saliva-mediated exposure.

Three patients with EBV-HLH associated with presumed primary EBV infection were identified. In all three patients, there was no personal or family history of recurrent infections or HLH suggestive of familial HLH (fHLH) or underlying immune disorders. Immunoglobulin levels were within the normal range. Whole-body computed tomography (CT) showed splenomegaly, which is defined as a splenic oblique coronal length exceeding 12 cm (9), but there were no mass lesions suggestive of lymphoma or other malignancies. Bone marrow examination showed hemophagocytosis or macrophage proliferation, but morphology and flow cytometry did not show distinct abnormal lymphoid population. Cytogenetic analysis showed a normal karyotype, and no clonal immunoglobulin heavy-chain (IgH) or T-cell receptor (TCR) gene rearrangement was detected in any patient. The detailed clinical courses of the three patients are described below. HLA screening and preparation for possible HSCT were carried out in case of inadequate disease control, but HSCT was not pursued because all three patients showed clinical improvement, and no HLH recurrence was observed during the available follow-up period.

### 2.1 Case 1

Case 1 was of a 19-year-old male. He had fever and sore throat with pharyngeal exudates. Physical examination revealed hepatosplenomegaly but no superficial lymphadenopathy nor rash. Laboratory examination revealed neutropenia, liver dysfunction, hyperferritinemia, and elevated soluble interleukin-2 receptor (sIL-2R). EBV serology showed anti-VCA IgM and IgG positivity and anti-EBNA negativity, and whole-blood EBV-DNA was 6.5 log IU/mL. These findings, acute clinical presentation and a history compatible with saliva-mediated exposure determined presumed primary EBV infection.

Bone marrow examination showed hemophagocytosis and EBV-positive cells. The EBV-infected cell phenotype could not be determined definitively because of the limited number of cells. Flow cytometry did not identify distinct abnormal lymphoid population. fHLH -associated genes were screened at Kazusa DNA Research Institute using a next-generation sequencing assay of protein-coding exons and flanking splice-site regions. No pathogenic variants were identified in *PRF1, UNC13D, STX11, STXBP2, FAAP24, SLC7A7, LYST, RAB27A, AP3B1, AP3D1, SH2D1A, XIAP (BIRC4), RHOG, CDC42, HAVCR2, NLRC4,* or *MVK*.

The patient's clinical course is shown in Figure 1. Dexamethasone and cyclosporine A were administered before the transfer to our institution, and etoposide was added according to the HLH-2004 protocol. Because he showed rapid clinical improvement after HLH-directed therapy and remained free of HLH recurrence, HSCT was not pursued. EBV-DNA became undetectable on day 190 but subsequently fluctuated at low levels and was 3.1 log IU/mL at the final follow-up on day 345 without clinical evidence of HLH recurrence.

**Figure 1:**
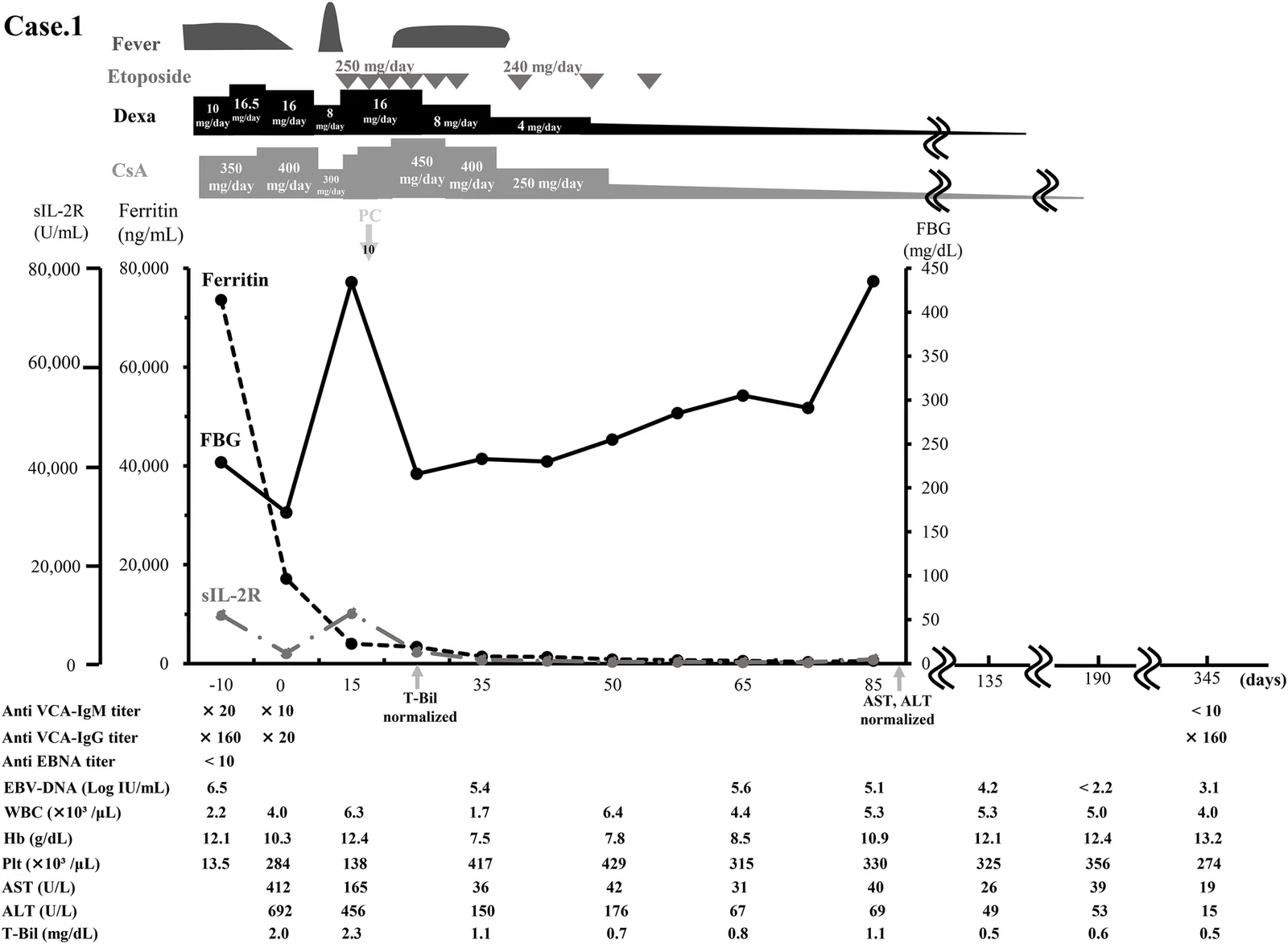
Clinical course of case 1. Dexa, dexamethasone; CsA, cyclosporine A; FBG, fibrinogen; sIL-2R, soluble interleukin-2 receptor; PSL, prednisolone; PC, platelet concentrate; WBC, white blood cell; Hb, hemoglobin; Plt, platelet.

### 2.2 Case 2

Case 2 was of a 26-year-old female. She developed fever and sore throat. Physical examination revealed hepatosplenomegaly but no superficial lymphadenopathy. Laboratory examination revealed pancytopenia with atypical lymphocytosis, liver dysfunction, hyperferritinemia, and elevated sIL-2R. EBV serology showed anti-VCA IgM and IgG positivity and anti-EBNA negativity, and whole-blood EBV-DNA was 5.3 log IU/mL. She was transferred to our hospital. Primary EBV infection was considered based on a history of saliva exposure.

Bone marrow aspiration showed hemophagocytosis. Given the clinical findings and EBV serology, she was diagnosed with HLH associated with presumed primary EBV infection. EBV-infected cell analysis was performed using peripheral blood mononuclear cells (PBMCs). The results are shown in Table 1. PBMCs were separated into lymphocyte fractions using antibody-conjugated magnetic beads (Miltenyi Biotec, Bergisch Gladbach, Germany), and EBV-DNA was quantified in each fraction (10). As shown in Table 1, EBV-DNA was detectable in CD8-positive T cells. We considered this finding compatible with transient CD8-positive T-cell infection that can occur during infectious mononucleosis.

**Table 1:** EBV-DNA levels in peripheral blood cell fractions in case 2.

Cell fraction	EBV-DNA load
Whole blood	8.2 × 10^4^ copies/*μ*g DNA
Plasma	1.2 × 10^6^ copies/mL
CD19^+^ cells	ND
CD4^+^ cells	ND
CD8^+^ cells	9.5 × 10^3^ copies/μg DNA
CD56^+^ cells	ND
Other cells	ND

ND, not detected.

The clinical course is shown in Figure 2. HLH-94-based therapy was initiated before the transfer to our institution. After the transfer, leukocyte and platelet counts recovered, and fever resolved promptly. Dexamethasone was tapered because there was no clinical evidence of persistent or recurrent HLH. EBV-DNA remained detectable throughout long-term follow-up at approximately 3.0 log IU/mL on average, below the diagnostic cutoff for CAEBV. Despite persistent EBV-DNA positivity, HLH did not recur. Approximately two years after diagnosis, her platelet count gradually decreased, but she did not fulfill the diagnostic criteria for recurrent HLH. CT showed no new splenic enlargement or findings suggestive of lymphoma, and platelet-associated IgG (PA-IgG) was positive. She was therefore considered to have immune thrombocytopenia and was managed by observation without specific treatment. She remained free of HLH recurrence through day 1,067.

**Figure 2:**
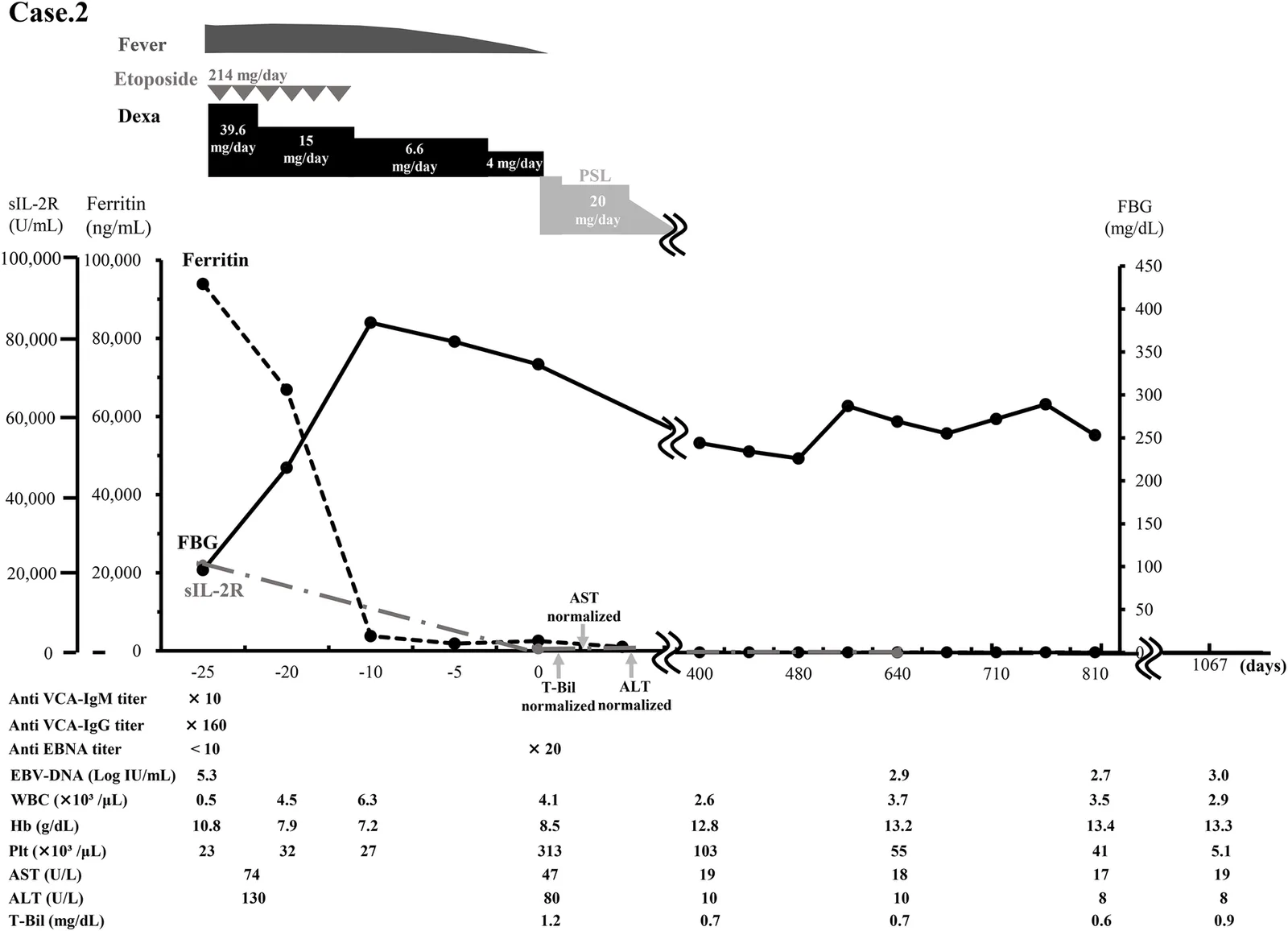
Clinical course of case 2. Dexa, dexamethasone; PSL, prednisolone; FBG, fibrinogen; sIL-2R, soluble interleukin-2 receptor; PC, platelet concentrate; FFP, fresh frozen plasma; PRBC, packed red blood cells; WBC, white blood cell; Hb, hemoglobin; Plt, platelet.

### 2.3 Case 3

Case 3 was of a 23-year-old male. He presented with persistent fever. Upon admission to our institution, physical examination revealed jaundice, but no superficial lymphadenopathy. Laboratory examination revealed pancytopenia, severe liver dysfunction, renal dysfunction, pleural effusion with respiratory failure, and hepatosplenomegaly. EBV serology showed anti-VCA IgM and IgG positivity and anti-EBNA negativity, and whole-blood EBV-DNA was 6.7 log IU/mL. Together with the acute clinical presentation and a history compatible with saliva-mediated exposure, these findings supported presumed primary EBV infection.

Bone marrow examination revealed marked macrophage proliferation with hemophagocytosis. EBV-infected cell analysis of the bone marrow demonstrated EBV infection in CD3-positive T cells. As mentioned earlier, transient EBV infection on T-cell can occur during infectious mononucleosis.

The clinical course is shown in Figure 3. HLH-94-based treatment with dexamethasone and etoposide was started. The initial etoposide dose was reduced because of severe hepatic impairment. The patient required mechanical ventilation and renal replacement therapy during the acute phase. However, these conditions improved rapidly after starting the treatment. On day 40, EBV-DNA decreased from 6.7 log IU/mL at presentation to approximately 3.2 log IU/mL and EBV-infected cells were not detected in the bone marrow. Around day 65, during dexamethasone tapering, the patient developed thrombocytopenia following fever, accompanied by an increase of sIL-2R to 2,149 U/mL and an increase of EBV-DNA to 5.3 log IU/mL. Because EBV-HLH reactivation was clinically suspected, dexamethasone was temporarily increased to 2.5 mg/day (1.25 mg/m^2^/day), the dose specified in the HLH-2004 protocol at that time point. The clinical findings and laboratory abnormalities improved promptly, and EBV-DNA subsequently decreased without additional treatment intensification or HSCT. Dexamethasone was then tapered carefully. The patient remained free of further HLH recurrence through day 426.

**Figure 3:**
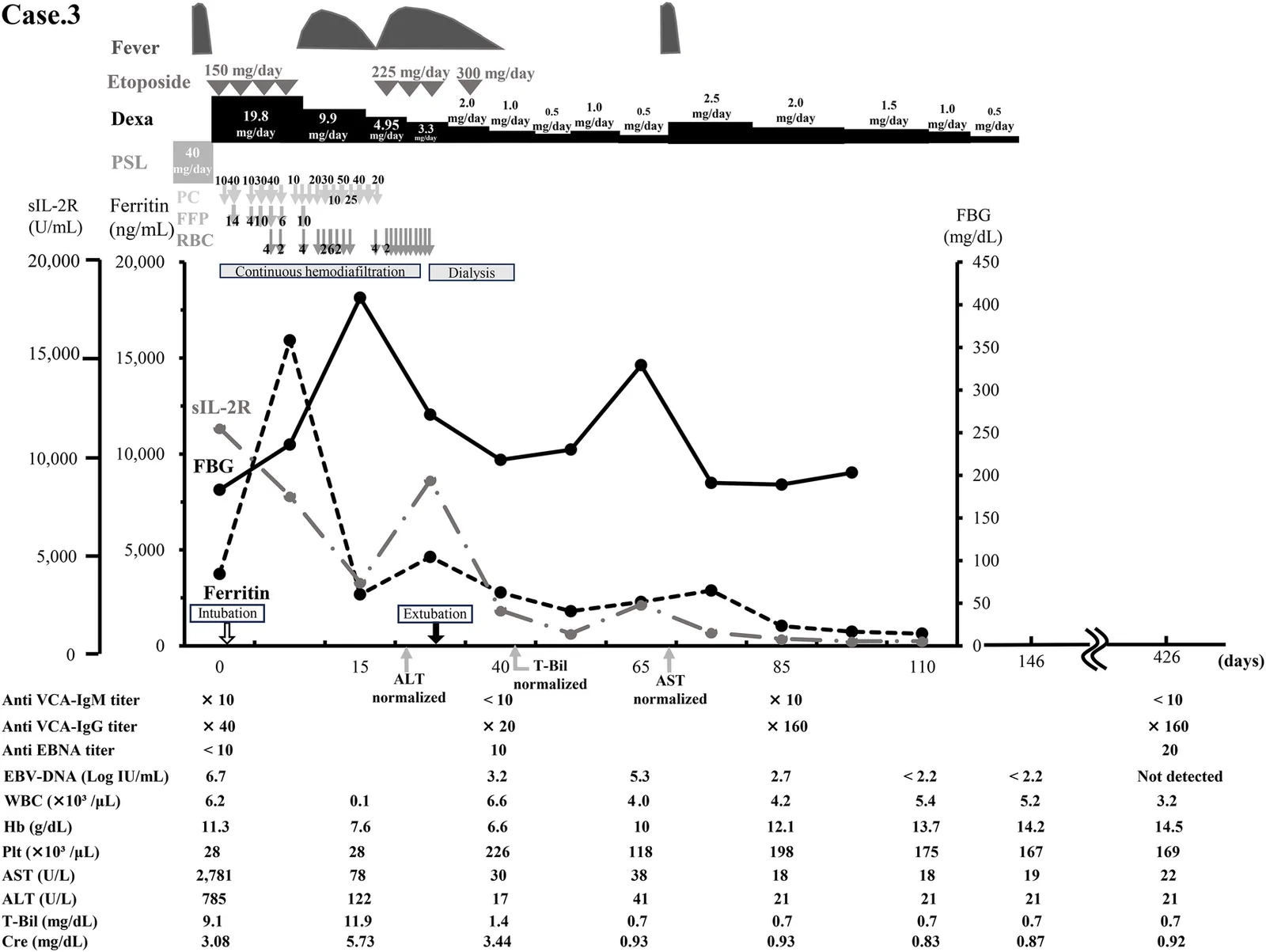
Clinical course of case 3. Dexa, dexamethasone; PSL, prednisolone; FBG, fibrinogen; sIL-2R, soluble interleukin-2 receptor; WBC, white blood cell; Hb, hemoglobin; Plt, platelet.

## 3 Discussion

EBV-HLH is a heterogeneous syndrome, and prognosis and treatment requirements, particularly the need for HSCT, differ according to the underlying disease context (5). In all three cases, HLH was associated with presumed primary EBV infection. All patients showed rapid improvement after HLH-directed therapy and remained free of HLH recurrence during the available follow-up period without HSCT. No clinical, pathological, or imaging findings supported EBV-positive lymphoma. However, genetic testing for fHLH was performed only in Case 1; in Cases 2 and 3, fHLH was considered unlikely based on the clinical findings alone. Similarly, comprehensive functional and genetic evaluations for inborn errors of immunity (IEI) were not performed in all patients. Thus, occult fHLH or IEI cannot be completely excluded.

Distinguishing primary EBV infection from other EBV-associated disorders remains challenging, particularly from CAEBV. The diagnosis of CAEBV requires exclusion of primary EBV infection (11, 12); nevertheless, EBV serology requires careful interpretation. Anti-VCA IgM antibodies may become undetectable after the early phase of primary infection. In addition, the absence of anti-EBNA antibodies alone does not necessarily indicate primary infection because anti-EBNA antibodies can be absent even in individuals with past EBV infection (13). Regarding EBV-DNA, its positivity alone cannot distinguish primary EBV infection from CAEBV. Moreover, EBV infection of T cells may be observed transiently during acute primary EBV infection (14, 15). In our cases, EBV infection of T cells was detected in Cases 2 and 3 during the early phase of the disease. The major clinical difference between these conditions is their subsequent course. Primary EBV infection may have a favorable outcome in patients without an underlying disorder, whereas CAEBV is generally progressive and may follow a fatal course if HSCT is not performed. In a nationwide Japanese survey, no long-term survivors were observed among patients who did not undergo HSCT (16). Longitudinal follow-up is essential to distinguish presumed primary EBV infection from CAEBV.

An important observation in this series was that EBV-DNA remained detectable or fluctuated after clinical remission without HLH recurrence. In Case 2, EBV-DNA persisted at approximately 3.0 log IU/mL for a prolonged period without recurrence of HLH. In Case 3, EBV-DNA transiently increased during dexamethasone tapering, accompanied by fever, thrombocytopenia, and an increase in sIL-2R, which led to temporary escalation of dexamethasone. The patient improved promptly and was subsequently managed without further treatment intensification or HSCT. These findings suggest that EBV-DNA levels after clinical remission alone should not be used as an indicator of disease activity. Rather, longitudinal clinical assessment should also be part of the determinators of the need for treatment intensification or HSCT. Recent studies emphasize the importance of clinical response and disease context when considering HSCT in EBV-HLH. La Rosée et al. recommend that allogeneic HSCT should be considered mainly for patients with refractory or recurrent disease, CAEBV, or underlying familial or genetic disorders (17). Yao et al. reported that patients who achieve complete remission after initial therapy may be managed with a wait-and-see strategy, whereas those with partial remission should be considered for HSCT (18). These concepts are consistent with our clinical approach. HSCT was not pursued in the present patients because they achieved prompt clinical improvement and remained free of HLH recurrence during follow-up.

Our study has several limitations. The number of cases is small, and the study was retrospective. The diagnostic evaluation was not uniform across the three patients. Lymph node biopsy was not performed because no clinically significant lymphadenopathy or mass lesions were identified. Comprehensive immunohistochemical analyses were not performed. Genetic testing for fHLH was performed only in Case 1. Therefore, occult fHLH, inborn errors of immunity, CAEBV, or EBV-positive lymphoma cannot be completely excluded in all patients.

In summary, these three cases suggest that EBV-DNA may remain detectable or fluctuate even after clinical remission in adults with HLH associated with presumed primary EBV infection. EBV-DNA positivity alone should not determine the indication for HSCT. Instead, the indication for HSCT should be determined on the basis of longitudinal clinical observation and repeated evaluation for CAEBV, EBV-positive lymphoma, fHLH, and underlying immunodeficiency.

## 4 Patient perspective

Patient perspectives were not collected in a structured manner because this was a retrospective case series.

## Data Availability

The datasets presented in this article are not readily available because they contain potentially identifiable patient information from a small case series. Sharing data is restricted to protect patients' privacy in accordance with institutional ethics requirements. Requests to access the datasets should be directed to the corresponding author, Ayako Arai, ara.hema@marianna-u.ac.jp.
